# Supplementary material for: Survival and lung function decline in patients with definite, probable and possible idiopathic pulmonary fibrosis treated with pirfenidone
Source: PLoS One. 2022 Sep 1;17(9):e0273854. doi: 10.1371/journal.pone.0273854 (PMC9436039; doi:10.1371/journal.pone.0273854)

S1 Fig. Flow diagram of patients participating in the study and their availability for performed analyses

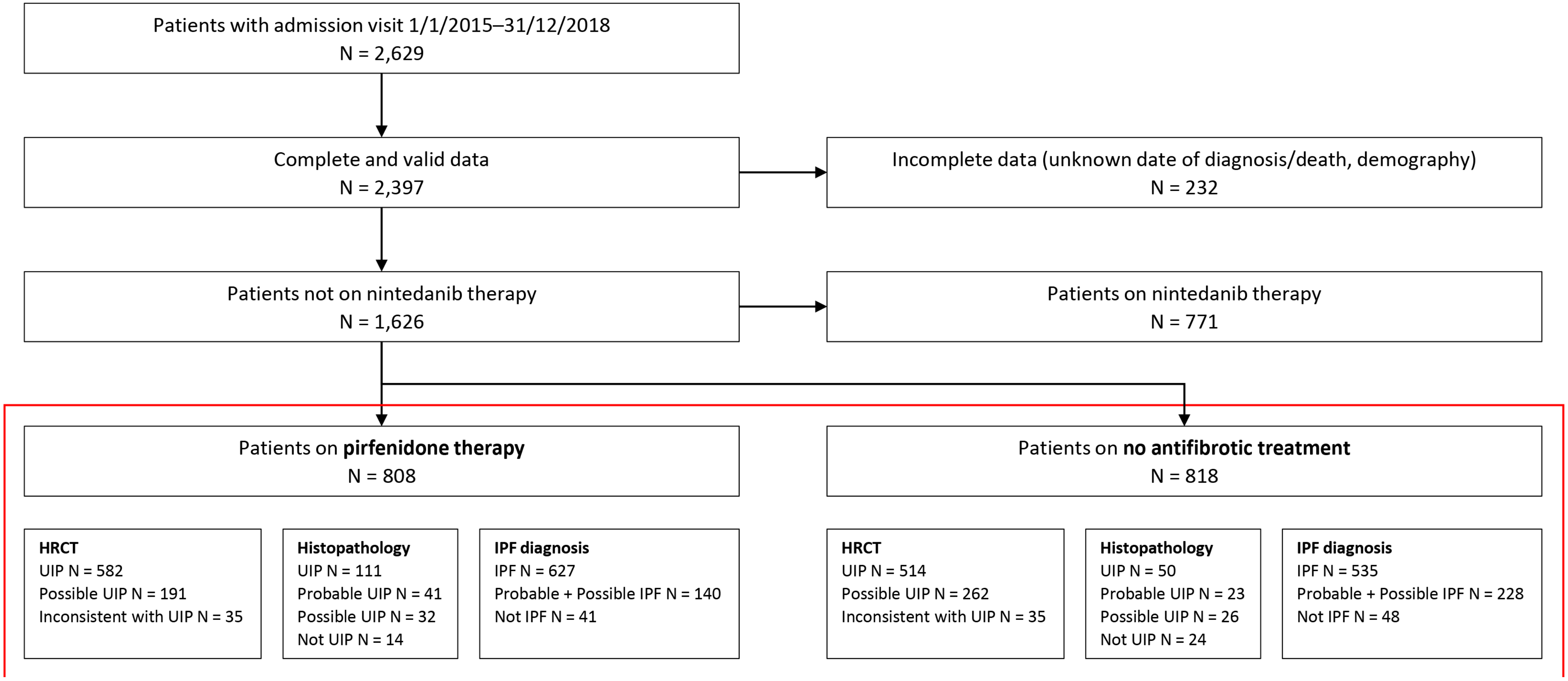

Supplement: S1 Fig — (PDF) [file pone.0273854.s001.pdf]
