# Supplementary material for: Survival and lung function decline in patients with definite, probable and possible idiopathic pulmonary fibrosis treated with pirfenidone
Source: PLoS One. 2022 Sep 1;17(9):e0273854. doi: 10.1371/journal.pone.0273854 (PMC9436039; doi:10.1371/journal.pone.0273854)
Supplement: S2 Fig — (PDF) [file pone.0273854.s002.pdf]

S2 Fig. Overall survival in diagnostic subgroups

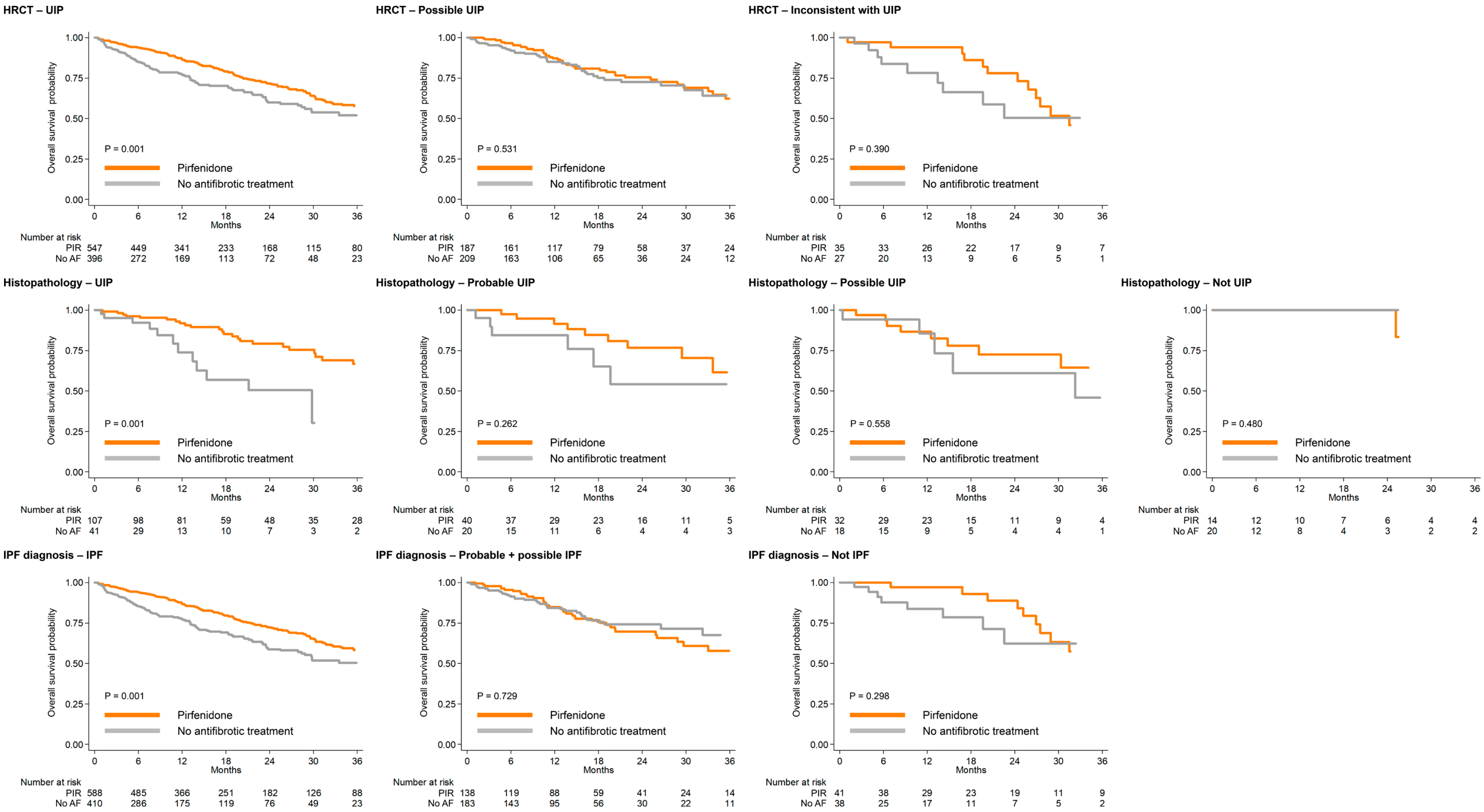

Interpretation: The UNADJUSTED survival analysis indicated differences in overall survival rates between the *pirfenidone group* and the *no antifibrotic treatment group* in most of the diagnostic subgroups, particularly in those with higher diagnostic certainty and higher number of patients. Please refer to Table 2 in the main article for adjusted analysis.
