# Supplementary material for: Survival and lung function decline in patients with definite, probable and possible idiopathic pulmonary fibrosis treated with pirfenidone
Source: PLoS One. 2022 Sep 1;17(9):e0273854. doi: 10.1371/journal.pone.0273854 (PMC9436039; doi:10.1371/journal.pone.0273854)
Supplement: S4 Fig — (PDF) [file pone.0273854.s004.pdf]

S4 Fig. Progression-free survival in diagnostic subgroups

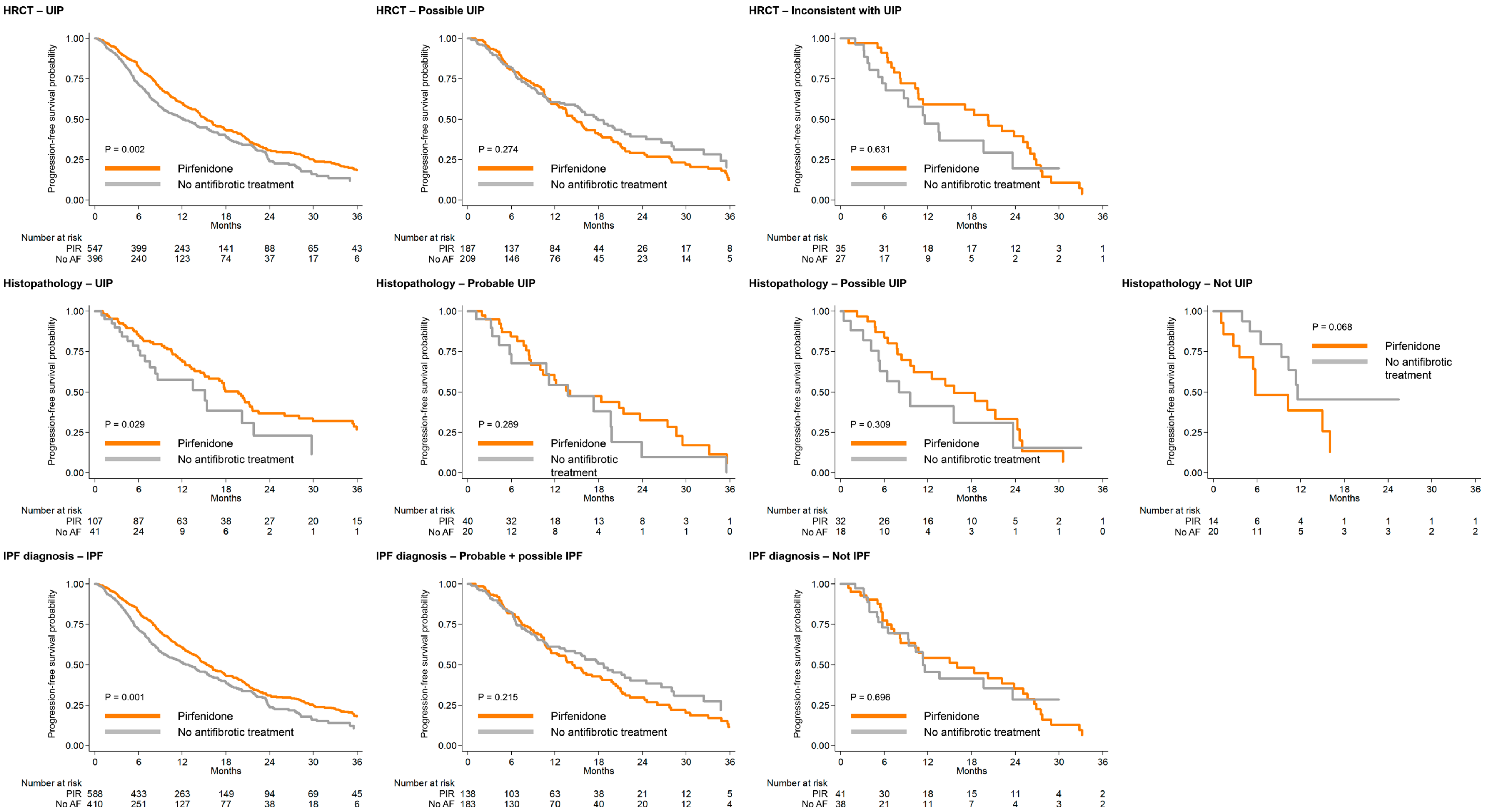

Interpretation: The UNADJUSTED survival analysis indicated differences in progression-free survival rates between the *pirfenidone group* and the *no antifibrotic treatment group* in most of the diagnostic subgroups, particularly in those with higher diagnostic certainty and higher number of patients. Please refer to Table 2 in the main article for adjusted analysis.
