# Supplementary material for: Survival and lung function decline in patients with definite, probable and possible idiopathic pulmonary fibrosis treated with pirfenidone
Source: PLoS One. 2022 Sep 1;17(9):e0273854. doi: 10.1371/journal.pone.0273854 (PMC9436039; doi:10.1371/journal.pone.0273854)
Supplement: S5 Fig — (PDF) [file pone.0273854.s005.pdf]

S5 Fig. Progression-free survival of patients treated with pirfenidone in diagnostic subgroups

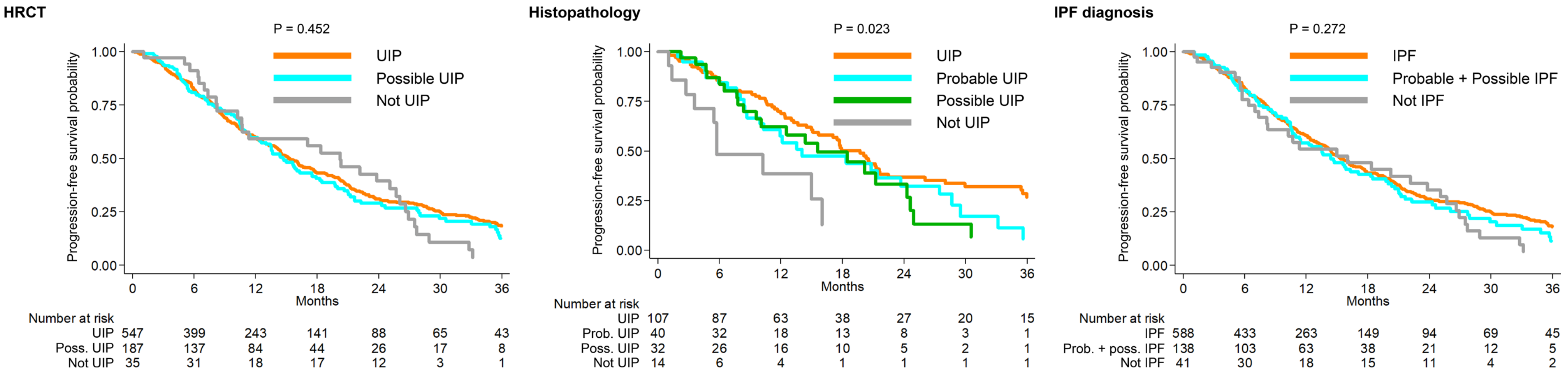

Interpretation: The UNADJUSTED survival analysis did not indicate differences in overall survival rates between the diagnostic subgroups in the *pirfenidone group*, with a possible exception for the less frequent “Not UIP” subgroup as shown by histopathology. Please refer to Table 2 in the main article for adjusted analysis.
