# Supplementary material for: Survival and lung function decline in patients with definite, probable and possible idiopathic pulmonary fibrosis treated with pirfenidone
Source: PLoS One. 2022 Sep 1;17(9):e0273854. doi: 10.1371/journal.pone.0273854 (PMC9436039; doi:10.1371/journal.pone.0273854)
Supplement: S2 Appendix — Pirfenidone Effectiveness In Idiopathic Pulmonary Fibrosis With Different Radiologic Patterns. (PDF) [file pone.0273854.s007.pdf]

ATS 2020 Where today's science meets tomorrow's care™ INTERNATIONAL CONFERENCE May 15 - May 20 | PHILADELPHIA

Print this Page for Your Records Close Window

Control/Tracking Number: 2020-S-11920-ATS
Activity: Scientific Abstract
Current Date/Time: 10/30/2019 4:10:06 AM

Pirfenidone Effectiveness In Idiopathic Pulmonary Fibrosis With Different Radiologic Patterns

Author Block: M. Vasakova[1], M. Sterclova[1], N. Mogulkoc[2], K. Lewandowska[3], V. Müller[4], M. Hajkova[5], J. Tekavec-Trkanjec[6], M. J. Studnicka[7], D. Jovanovic[8], N. Y. Stoeva[9], S. Littnerova[10], L. Dusek[10];
[1]Department of Respiratory Medicine, Thomayer Hospital, Praha, Czech Republic, [2]Istanbul Medikal Saglik Ve Yayinclinik Millet, 34104 CAPA- Istanbul, Turkey, [3]1st Department of Pulmonary Diseases, Institute of Tuberculosis and Lung Diseases, Warszawa, Poland, [4]Semmelweis University, Budapest, Hungary, [5]University Hospital Bratislava, Bratislava, Slovakia, [6]University Hospital Dubrava, Zagreb, Croatia, [7]Univ Klinik Pneumology, Salzburg, Austria, [8]Thoracic Oncology and ILD, University Hospital of Pulmonology, Belgrade, Serbia, [9]Pulmonary dept., Tokuda hospital Sofia, Sofia, Bulgaria, [10]Masaryk University, Brno, Czech Republic.

Abstract:
Aim: To assess effectiveness of pirfenidone among patients with differing degrees of diagnostic certainty of radiologic patterns according ATS/ERS/JRS/ALAT guidelines 2011.
Patients and methods: 844 IPF patients treated by pirfenidone (PIR) and 894 with other (no antifibrotic) IPF treatment (OT) were enrolled to retrospective study from the EMPIRE registry. Both groups were stratified to radiologic subgroups UIP, Possible UIP, and Inconsistent with UIP. FVC, DLCO and 6MWD were investigated at baseline and then after 6,12,18 and 24 months(M). Effectiveness was evaluated by change in lung function over time and proportion of the patients with major progressive events (>10% absolute decline in % predicted FVC from baseline, >15% absolute decline in % predicted DLCO from baseline, >50 m decline in 6MWD from baseline) in PIR versus OT group.
Results: There were 600, 205 and 39 PIR patients and 565, 293 and 36 OT in the UIP, Possible UIP and Inconsistent with UIP group. FVC decline>10% was observed in 38 (8.4%) PIR and 30 (14.8%) OT patients at 6 M(p=0.016), 37(11.6%) and 21(14.0%) (NS) at 12 M, 39(17.1%) and 18(16.7%) (NS) at 18 M, 38(25.2%) and 13(20.0%) (NS) at 24 M. The difference in proportion of patients with FVC decline>10% was only in the UIP group at 6M: 27 PIR(8.7%) vs 22(19.8%) OT patients (p=0.003). DLCO decline>15% was observed in 44(10.0%) PIR and in 25(12.7%) OT patients at 6M(NS), 41(12.9%) and 15(10.5%) (NS) at 12M, 33(14.5%) and 15(14.2%) (NS) at 18M, 33(22.1%) and 12(19.0%) (NS) at 24M. The difference in proportion of patients with DLCO decline>15% observed only in the UIP group at 6M: 28 PIR(9.3%) vs 18(16.8%) OT patients (p=0.041). 6MWD decline>50 m was observed in 33 (15.6%) PIR and in 11 (11.7%) OT patients at 6M(NS), 34(24.6%) and 12(17.9%) (NS) at 12M, 32(38.6%) and 10(17.9%) (p=0.008) at 18M, 22(44.0%) and 3(9.7%) (p=0.001) at 24M. In HRCT subgroups, a difference in decline of 6MWD was in the UIP (18 and 24M) and in the Possible UIP (12 and 24M).
Conclusion: The decline of FVC and DLCO was similar in all HRCT subgroups, except UIP at 6M, where significantly less UIP-PIR patients had significant decline of FVC and DLCO than UIP-OT. The significant decline of 6MWD was more frequently seen in OT group at 18 and 24 M than in PIR and less patients with PIR-UIP and Possible UIP group had significant decline of 6MWD at 18 and 24M compared to OT.
:

Category (Complete): 12. Diffuse Parenchymal Lung Diseases: ILD, Sarcoidosis, IPF, LAM -> Adult -> Clinical Studies /Clinical Problems (CP)
Presentation Preference (Complete): Either Poster or Oral
Abstract Affirmations (Complete):
Basic Science Core Track: No
Related to Health Equality?: No
Rare Lung Disease Guide: No
LMIC: No
Funded by : The study was supported by a research grant by Roche; the EMPIRE registry is supported by Boehringer Ingelheim and Roche
I agree to the Author Acknowledgement Statement : True
I agree to the Redundancy Statement : True
I agree to the Prior Publication Statement : True
I agree to the Terms of Use : True

Presenter Affirmations (Complete):
Please select your primary ATS Assembly affiliation from the list below. Select "None" if you do not have an assembly affiliation OR are a non-member.: Clinical Problems (CP)
Nursing Degree?: No
If No, Do any other authors on this abstract have a nursing degree of any kind?: No
First/Second Year Fellow?: No
Student or in training?: No
Early Stage Investigator?: No
New Submitter?: No
Scholarship Applicant?: No
Publish Presenter Email with Abstract?: No
Do you agree?: Yes

Clinical Trial Affirmation (Complete):
1. Is this abstract reporting results from an NIH-defined Clinical Trial?

If yes, what phase(s) is/are this Clinical Trials. Select all that apply.: No

Status: Finalized

American Thoracic Society
25 Broadway, New York 10004-1012
P: 212-315-8600
F: 212-315-8653

ATS Technical Support
Email: ats@support.ctimeetingtech.com
Phone: 217-398-1792
Technical Support is available Monday-Friday, 8am-5pm Central Time.

Feedback

Powered by cOASIS, The Online Abstract Submission and Invitation System SM
© 1996 - 2019 CTI Meeting Technology All rights reserved. Privacy Policy
