# Supplementary material for: Survival and lung function decline in patients with definite, probable and possible idiopathic pulmonary fibrosis treated with pirfenidone
Source: PLoS One. 2022 Sep 1;17(9):e0273854. doi: 10.1371/journal.pone.0273854 (PMC9436039; doi:10.1371/journal.pone.0273854)
Supplement: S1 Table — (PDF) [file pone.0273854.s008.pdf]

S1 Table. Baseline characteristics of patients with pirfenidone in IPF diagnostic subgroups (IPF x probable + possible IPF x not IPF). Data are given as mean (±SD) or N (%)

		**IPF**<br>**N = 627**	**Probable + possible IPF**<br>**N = 140**	**Not IPF**<br>**N = 41**
Demographics	Men	489 (78.0%)	98 (70.0%)	22 (53.7%)
	Age (years)	67.8 (±8.9)	68.0 (±8.0)	63.7 (±9.0)
	BMI	28.3 (±4.3)	28.5 (±4.7)	30.3 (±4.9)
Smoking	Never-smokers	213 (34.0%)	54 (38.6%)	22 (53.7%)
	Ex-smokers	85 (13.6%)	13 (9.3%)	3 (7.3%)
	Current smokers	329 (52.5%)	73 (52.1%)	16 (39.0%)
HRCT pattern	Definite UIP	575 (91.7%)	0 (0.0%)	7 (17.1%)
	Possible UIP	52 (8.3%)	133 (95.0%)	6 (14.6%)
	Inconsistent with UIP	0 (0.0%)	7 (5.0%)	28 (68.3%)
Histopathology	UIP	104 (16.6%)	7 (5.0%)	0 (0.0%)
	Probable UIP	38 (6.1%)	0 (0.0%)	3 (7.3%)
	Possible UIP	14 (2.2%)	17 (12.1%)	1 (2.4%)
	Not UIP	0 (0.0%)	0 (0.0%)	14 (34.1%)
	Not performed	471 (75.1%)	116 (82.9%)	23 (56.1%)
IPF diagnosis	IPF	627 (100.0%)	0 (0.0%)	0 (0.0%)
	Probable + possible IPF	0 (0.0%)	140 (100.0%)	0 (0.0%)
	Not IPF	0 (0.0%)	0 (0.0%)	41 (100.0%)
Comorbidities	Number of comorbidities	3.56 (2.03)	3.59 (2.04)	3.78 (1.61)
	Heart and vascular	465 (74.2%)	98 (70.0%)	33 (80.5%)
	Pulmonary	165 (26.3%)	65 (46.4%)	21 (51.2%)
	Gastrointestinal	377 (60.1%)	75 (53.6%)	31 (75.6%)
	Urogenital	113 (18.0%)	18 (12.9%)	2 (4.9%)
	Cancer	31 (4.9%)	10 (7.1%)	4 (9.8%)
Comedication	At least one comedication	531 (84.7%)	121 (86.4%)	33 (80.5%)
	Number of comedications	2.83 (2.13)	2.76 (2.26)	2.49 (2.24)
	Beta-blockers	200 (31.9%)	34 (24.3%)	8 (19.5%)
	ACEI	168 (26.8%)	33 (23.6%)	5 (12.2%)
	Aspirin	174 (27.8%)	33 (23.6%)	10 (24.4%)
	Statins	181 (28.9%)	27 (19.3%)	5 (12.2%)
	Diuretics	121 (19.3%)	24 (17.1%)	6 (14.6%)
IPF treatment	Pharmacological	627 (100.0%)	140 (100.0%)	41 (100.0%)
	Rehabilitation	153 (24.4%)	31 (22.1%)	7 (17.1%)
	LTOT	131 (20.9%)	29 (20.7%)	10 (24.4%)
	Lung transplantation	85 (13.6%)	12 (8.6%)	6 (14.6%)
Pharmacological treatment (non-AF)	N-acetylcysteine	98 (15.6%)	23 (16.4%)	2 (4.9%)
	Proton pump inhibitors	117 (18.7%)	27 (19.3%)	10 (24.4%)
	Systemic corticosteroids	76 (12.1%)	21 (15.0%)	6 (14.6%)
	Azathioprine	19 (3.0%)	4 (2.9%)	3 (7.3%)
	Other cytostatic	5 (0.8%)	3 (2.1%)	2 (4.9%)
Lung functions at the therapy initiation ± 3 months	FVC predicted (%)	74.5 (±16.0) / 427[1]	72.3 (±16.6) / 104[1]	68.6 (±15.0) / 22[1]
	DLCO predicted (%)	47.2 (±14.9) / 402[1]	44.9 (±12.9) / 97[1]	45.8 (±14.7) / 19[1]
GAP index	I	195 (41.1%)	48 (41.0%)	15 (53.6%)
	II	220 (46.3%)	57 (48.7%)	12 (42.9%)
	III	60 (12.6%)	12 (10.3%)	1 (3.6%)
NYHA	I	23 (4.9%)	5 (4.3%)	1 (3.1%)
	II	264 (56.8%)	58 (49.6%)	13 (40.6%)
	III	167 (35.9%)	52 (44.4%)	17 (53.1%)
	IV	11 (2.4%)	2 (1.7%)	1 (3.1%)

[1] *Number of patients for whom the baseline value of FVC predicted or DLCO predicted was available*
