# Supplementary material for: Survival and lung function decline in patients with definite, probable and possible idiopathic pulmonary fibrosis treated with pirfenidone
Source: PLoS One. 2022 Sep 1;17(9):e0273854. doi: 10.1371/journal.pone.0273854 (PMC9436039; doi:10.1371/journal.pone.0273854)
Supplement: S2 Table — (PDF) [file pone.0273854.s009.pdf]

S2 Table. Univariate hazard ratio for mortality and progression (N = 1,402)

	**Mortality HR (95% CI)**	**P**	**Progression HR (95% CI)**	**P**
Pirfenidone	0.714 (0.579; 0.880)	**0.002**	0.866 (0.753; 0.995)	**0.043**
Height	0.983 (0.972; 0.994)	**0.002**	0.995 (0.988; 1.002)	0.178
Age	1.021 (1.009; 1.033)	**0.001**	1.015 (1.007; 1.023)	**<0.001**
Gender – male	1.257 (0.943; 1.674)	0.119	1.168 (0.983; 1.387)	0.078
FVC (L) at baseline	0.597 (0.509; 0.700)	**<0.001**	0.878 (0.802; 0.961)	**0.005**
NYHA – II	2.615 (1.149; 5.950)	**0.022**	1.481 (1.072; 2.044)	**0.017**
NYHA – III	6.816 (3.012; 15.422)	**<0.001**	1.930 (1.390; 2.680)	**<0.001**
NYHA – IV	11.988 (4.346; 33.066)	**<0.001**	1.584 (0.831; 3.019)	0.162
