# Supplementary material for: Survival and lung function decline in patients with definite, probable and possible idiopathic pulmonary fibrosis treated with pirfenidone
Source: PLoS One. 2022 Sep 1;17(9):e0273854. doi: 10.1371/journal.pone.0273854 (PMC9436039; doi:10.1371/journal.pone.0273854)
Supplement: S3 Table — (PDF) [file pone.0273854.s010.pdf]

S3 Table. Multivariate hazard ratio for mortality and progression (adjusted for age, sex, height, NYHA and FVC at baseline, N = 1,128)

	**Mortality HR (95% CI)**	**P**	**Progression HR (95% CI)**	**P**
Pirfenidone	0.749 (0.575; 0.976)	**0.032**	1.037 (0.880; 1.222)	0.666
Height	0.989 (0.969; 1.010)	0.301	1.004 (0.992; 1.017)	0.517
Age	1.009 (0.994; 1.025)	0.235	1.005 (0.996; 1.014)	0.289
Gender – male	2.681 (1.788; 4.020)	**<0.001**	1.296 (1.016; 1.655)	**0.037**
FVC (L) at baseline	0.530 (0.421; 0.666)	**<0.001**	0.827 (0.726; 0.941)	**0.004**
NYHA – II	1.848 (0.799; 4.272)	0.151	1.324 (0.944; 1.857)	0.104
NYHA – III	4.037 (1.738; 9.376)	**0.001**	1.759 (1.234; 2.506)	**0.002**
NYHA – IV	7.532 (2.536; 22.375)	**<0.001**	1.484 (0.731; 3.011)	0.274
