# Supplementary material for: Survival and lung function decline in patients with definite, probable and possible idiopathic pulmonary fibrosis treated with pirfenidone
Source: PLoS One. 2022 Sep 1;17(9):e0273854. doi: 10.1371/journal.pone.0273854 (PMC9436039; doi:10.1371/journal.pone.0273854)
Supplement: S4 Table — (PDF) [file pone.0273854.s011.pdf]

S4 Table. Difference of annual DLCO decline according to diagnostic subgroups (adjusted for age, sex, height, NYHA and absolute FVC at baseline)

	No. of patients (pirfenidone)	DLCO annual rate – pirfenidone (95% CI) (mmol/kPa/min/yr)	No. of patients (no antifibrotics)	DLCO annual rate – no antifibrotics (95% CI) (mmol/kPa/min/yr)	P[1]	P for interaction[2]
Total	505	-0.187 (-0.396; 0.023)	421	-0.116 (-0.355; 0.122)	0.665	
HRCT						
UIP	344	-0.167 (-0.425; 0.092)	257	-0.177 (-0.494; 0.140)	0.962	
Possible UIP	139	-0.114 (-0.501; 0.272)	144	-0.062 (-0.445; 0.321)	0.851	0.620
Inconsistent with UIP	22	-0.916 (-1.905; 0.074)	20	-0.151 (-1.278; 0.975)	0.318	
Histopathology						
UIP	68	-0.306 (-1.106; 0.495)	26	0.355 (-1.221; 1.931)	0.464	
Probable UIP	27	-0.392 (-1.624; 0.840)	15	-0.810 (-2.347; 0.726)	0.678	0.734
Possible UIP	23	0.284 (-0.969; 1.537)	14	-0.360 (-1.775; 1.056)	0.504	
Not UIP	9	-0.447 (-2.644; 1.749)	17	-0.421 (-1.785; 0.942)	0.984	
IPF diagnosis						
IPF	376	-0.138 (-0.381; 0.105)	267	-0.147 (-0.464; 0.170)	0.963	
Probable IPF	98	-0.198 (-0.663; 0.267)	130	-0.101 (-0.512; 0.309)	0.759	0.542
Not IPF	31	-0.958 (-1.929; 0.013)	24	-0.230 (-1.015; 0.556)	0.253	

[1] Statistical significance of difference of annual rate of decline between patients with pirfenidone vs no antifibrotic treatment

[2] Statistical significance of impact of category on difference of annual rate of decline tested by LR test; higher P value (above 0.05) indicates that the possible effect of pirfenidone therapy (a difference between pirfenidone and no antifibrotic treatment) is similar across all diagnostic subgroups
