# Supplementary material for: Survival and lung function decline in patients with definite, probable and possible idiopathic pulmonary fibrosis treated with pirfenidone
Source: PLoS One. 2022 Sep 1;17(9):e0273854. doi: 10.1371/journal.pone.0273854 (PMC9436039; doi:10.1371/journal.pone.0273854)
Supplement: Attachment — Submitted filename: answers-reviewers.docx [file pone.0273854.s012.docx]

Dear Editor

We would like to thank you and the reviewer for the comprehensive review of our article. We have made changes to reflect readability and clarity of the paper as suggested.

## Editors' comments

We modified and transferred information about funding and acknowledgements as requested. We explained connection to the previous publication (ATS poster) in the cover letter.

Please note that a new Conflict of Interest statement of Veronika Müller has been added.

## Reviewers' comments

*1) Since probable/possible UIP are diagnosed as UIP in the latest guideline, the title of this manuscript is of little appeal to physicians managing IPF treatment.*

We agree that the stratification according to the diagnostic certainty with regard to applied treatment has currently become of lower importance. However, our paper demonstrates that this practice is justifiable and that the therapy likely works in all groups. Such evidence has been missing for pirfenidone. We therefore prefer to maintain the current title.

*2) In this study, how are patients who have been discontinued from pirfenidone treatment or who have been switched from nintedanib to pirfenidone?*

Described in Methods: “Patients followed in EMPIRE and receiving nintedanib at any time were excluded from analysis (see S1 Fig 1).” The “control” group contains only patients with no history of antifibrotic treatment. Figure legends and tables were corrected from “other treatment” to “no antifibrotic treatment” to be consistent with the rest of the paper.

*3) mention more deeply the difference from nintedanib treatment and how the results of this study affect when you are confused with nintedanib. There is a need to*

If the reviewer refers to switch from/to nintedanib, we agree that the nintedanib phase should not be included. This is why we excluded patients with previous nintedanib therapy from the analysis (since the final therapy outcome might be affected).

*4) Since nintedanib is indicated for PF-ILD, the chances of introducing pirfenidone are decreasing currently. The significance of the results of this study on the treatment of pirfenidone in such situations should be discussed more.*

This is a correct point, but we feel that it falls out of scope of our paper a bit. Furthermore, prescription of both antifibrotics differs from country to country, even within the EMPIRE registry; we therefore assume that pirfenidone remains an important option, at least for patients who do not tolerate the nintedanib therapy well.

*5) Do “other treatments” in Tables and Figures refer to “no antifibrotic treatment”? Does it also include nintedanib treatment as other treatment? It’s easy to get confused, so it’s better to unify them so that they are easy to understand.*

Yes, thank you, legends have been corrected as explained above.

*6) Please describe the difference in the onset of acute exacerbations between the pirfenidone group and the untreated group. It’s very important to discuss why the survival curves are different between these groups in this study. It is plausible that the prolongation effect of OS cannot be explained only by suppressing the decline in FVC by pirfenidone treatment.*

This is a reasonable assumption, but we found no difference in the onset of acute exacerbations between the pirfenidone and no antifibrotics group when analysing this dataset (median time to the first AE 5.38 months for PIR vs 5.48 months for no AF, P = 0.509). However, overall frequency of exacerbations was relatively low (60 events in the entire cohort of 1,600 patients).

*7) In the section of the results, “Overall survival and progression-free survival according to diagnostic certainly of IPF”, the authors should explain all data of the Figures in detail.*

We have added a brief interpretation and summary under each Figure in the supplementary material.

*You mentioned that there was no significant different effect of pirfenidone treatment on OS for the different diagnostic IPF subgroups in Table 2 and Sup. Fig. 2. In Table 2, it seems that there was a difference between diagnostic certainly of IPF distinct from Sup Fig. 2. It was hard to understand your results for general readers.*

Hopefully the interpretation under the supplementary figures helps. We also modified the main text referring to these outputs – Table 2 is an adjusted analysis in which differences between pirfenidone and no antifibrotics are assessed, while Suppl. Fig. 3 (originally Suppl. Fig. 2 in the reviewed version of the paper, see below) is an unadjusted analysis comparing pirfenidone patients only, without any involvement of patients with no antifibrotics.

*Further, you described the OS in patients treated with pirfenidone regarding Sup. Fig. 3. However, Sup. Fig. 3 demonstrates PFS, but not OS, in diagnostic subgroups.*

*Moreover, I cannot find Sup. Fig. 5 in the supplementary file.*

Apology for this inconvenience – the author responsible for the preparation of figures and supplementary material forgot to include Figure 1 in the supporting info document (a flow diagram of patients included in the analysis). Numbering of all supplementary figures therefore moved by one. The revised version includes the correct supplementary Figure 1 and numbering has been corrected.

*Please specify the definition of p1 and p2 in the Tables. Your presentation throughout the results including table capture is unfriendly to the readers.*

Added in headings and footnotes for better clarity.
