# Supplementary material for: Survival and lung function decline in patients with definite, probable and possible idiopathic pulmonary fibrosis treated with pirfenidone
Source: PLoS One. 2022 Sep 1;17(9):e0273854. doi: 10.1371/journal.pone.0273854 (PMC9436039; doi:10.1371/journal.pone.0273854)
Supplement: Attachment — Submitted filename: revisions PLOS.docx [file pone.0273854.s013.docx]

# Survival and lung function decline in patients with definite, probable and possible idiopathic pulmonary fibrosis treated with pirfenidone

## Revisions

Review Questions

2. Is the manuscript technically sound, and do the data support the conclusions?

Overall, the study is technically sound in its objective and methods; however, there is discordance between the numbers in the results section of the article and the tables, please see the revisions below and make the appropriate corrections.

5. Is the manuscript presented in an intelligible fashion and written in standard English?

There are multiple grammatical and syntax errors that, if corrected, will make the article easier to read and follow.

Revisions

1. Methods: “Patients with no observed event during follow-up (death, lung transplantation or progression of disease, see definition above) were censored at the date of the last visit to the registry, the date of last documented pirfenidone treatment or the date of loss to follow-up, whichever came last.” - It would be of value to know how patients that were started on pirfenidone and had to discontinue the medication early due to side effects were handled in the analysis. Eg. if patient was on pirfenidone for 1 month and then had to discontinue, was this patient still included in the pirfenidone arm? If there was a large proportion of patients like this, it would affect the interpretation of the results. Recommended addressing if this was considered and how it was handled, consider including the median duration of therapy (treatment with pirfenidone).
2. Table 1: report standard deviations with ±
3. Table 1: BMI and % of non-smokers reported in table do not match with what is written in the body of text.
4. Table 1: in the FVC and DLCO category, does the number in parenthesis represents standard deviation? If so, consider including the ± to make it clear. Currently, it is confusing because in the first column the % sign is in parenthesis (%).
5. Table 1: under smoking, is the P value representing the comparison between non-smokers? If so, then it should be placed in line with this row and not spanning the 3 rows. Similarly for HRCT pattern, Histopathology, IPF diagnosis, GAP index and dyspnoea.
6. Table 1: baseline FVC and DLCO do not match with the data on supplementary Table 4.
7. Supplementary fig 1 flow diagram has an error. The numbers under patients with pirfenidone therapy and IPF diagnosis do not add up to 808 and do not match with Table 1. Table 1 does contain appropriate numbers as they add up to n=808.
8. Table 2 and Table 3: caption 2 is confusing and misleading. It reads: “higher P value (above 0.05) indicates that the difference between pirfenidone and no antifibrotic treatment is consistent across all diagnostic subgroups,” this implies that there is a difference between the subgroups when the P value is above 0.05). Consider: higher P value (above 0.05) indicates that the difference between pirfenidone and no antifibrotic treatment in the different subgroups is not statistically significant.
9. Supp. Table 3. The total number of patients on pirfenidone and on no antifibrotic treatment are different compared to the ones on Table 3 (FVC decline), why is this? Did some patients not have DLCO recorded?
10. Discussion, lines 271-272: For clarification, in which study was median time to death not reached?

11. Discussion, lines 312-314: is there a reference for this statement?
12. Figure 2. The legend has been corrected to describe the groups as “pirfenidone group” and the “no antifibrotic treatment group”; however, under each individual image within figure 2, legend still reads PRI and OT. Same applies for Figure 1a,1b as well as S1 Fig 4. If keeping the abbreviation OT, please specify that it represents the no antifibrotic group.

Grammatical/syntax corrections

1. Methods: We followed patients in the multi-national European MultiPartner IPF Registry (EMPIRE) first seen **in between 2015 and 2018**.” – grammatical error, just remove “in” so that it reads first seen between 2015 and 2018.
2. Introduction: “One group of particular concern is IPF patients, who do not present with definite usual interstitial pneumonia (UIP) on HRCT, but demonstrate the radiological and/or histopathological pattern of probable/ possible UIP” - syntax error, remove comma after IPF patients
3. Results – Overall survival and progression-free survival according to diagnostic certainty of IPF: “although there seems to be less pronounced difference in crude survival curves for some subgroups (S1 Fig 2).” – this statement is missing an “a”- “although there seems to be **a** less pronounced difference in crude survival curves for some subgroups **in the unadjusted survival analysis** (S1 Fig 2).”
4. Results – Overall survival and progression-free survival according to diagnostic certainty of IPF: “Although the unadjusted comparison suggested a possible effect of pirfenidone on PFS (S1 Fig 4), with PFS in patients treated with pirfenidone rather similar among subgroups (S1 Fig 5), this was not confirmed by adjusted multivariate analysis (Table 2)” – This phrase can be made friendlier to the reader, consider -Although the unadjusted comparison suggested an a possible effect of pirfenidone on PFS (S1 Fig 4), **also observed among** subgroups (S1 Fig 5), this was not confirmed by adjusted multivariate analysis
5. Supplementary fig 1. Patients on/not on pirfenidone (or nintedanib) or patients not on pirfenidone (instead of with and without)
6. Line 87: should read: population, importance new evidence
7. Line 94: should read: January 1^st^ 2015 and December 31^st^ 2018 and followed through October 29^th^ 2019.
8. Line 96: should read: patient included in the EMPIRE registry
9. Line 98: should read: indicated, and a MDT discussion for final…
10. Line 108: remove word “onwards”
11. Line 163: significance was set “at” instead of set “on”
12. Line 196: authors use a hyphen (-) in no-antifibrotic treatment. No hyphen is used throughout the rest of the article. Consider removing it to stay consistent throughout the text.
13. Line 258-260: “Patient’s characteristics and the course of IPF were analyzed for those, who were treated with pirfenidone and compared to those receiving no antifibrotic treatment.” – this sentence does not read well. Consider: The clinical characteristics and course of IPF for patients receiving pirfenidone were compared to those of patients on no antifibrotic treatment.
14. Line 318: decrease in time 🡪 decrease over time
15. Line 320: patient with no antifibrotic treatment 🡪 patients on no antifibrotic treatment
16. Fig 1 demographics instead of demography
